# Determinants of Breastfeeding Practices and Its Association With Infant Anthropometry: Results From a Prospective Cohort Study in South India

**DOI:** 10.3389/fpubh.2020.492596

**Published:** 2020-10-14

**Authors:** Srinidhi Koya, Giridhara R. Babu, Deepa R, Veena Iyer, A. Yamuna, Eunice Lobo, Prafulla S, Sanjay Kinra, G. V. S. Murthy

**Affiliations:** ^1^DTA3 MSCA Research Fellow, School of Social Sciences, Humanities & Law, Teesside University, Middlesbrough, United Kingdom; ^2^Public Health Foundation of India and Wellcome Trust-DBT India Alliance Research Fellow in Public Health, New Delhi, India; ^3^Research Fellows, Indian Institute of Public Health Bangalore, Public Health Foundation of India, New Delhi, India; ^4^Indian Institute of Public Health Gandhinagar, Public Health Foundation of India, New Delhi, India; ^5^Indian Institute of Public Health Bangalore, Public Health Foundation of India, New Delhi, India; ^6^London School of Hygiene & Tropical Medicine & University College London Hospital, London, United Kingdom; ^7^Public Health Foundation of India, and International Center for Eye Health, Faculty of Infectious and Tropical Diseases, London School of Hygiene & Tropical Medicine, London, United Kingdom

**Keywords:** exclusive breastfeeding, supplementary feeding, wasting, under nutrition, South India

## Abstract

**Introduction:** Despite national efforts for promoting exclusive breastfeeding (EBF) during the first 6 months of the infants' life, breastfeeding rates are low in India. Evidence on the interference of supplementary food on optimal nourishment and growth of the infant has also been well-established. Our study was undertaken to assess the effect of breastfeeding practices on infant anthropometry and determine the various factors affecting breastfeeding practices.

**Methods:** A prospective cohort study - Maternal antecedents of adiposity and studying the transgenerational role of hyperglycemia and insulin (MAASTHI) was conducted at a tertiary care public hospital in Bengaluru, South India. From the consenting women, data such as obstetric history, infant feeding practices, anthropometry of mother and child, the psychosocial status of the women using the Edinburgh Postnatal Depression Scale (EPDS), was collected at baseline and subsequent follow-up: post-delivery and 14 weeks after birth. In this study, we analyzed data collected from April 2016 to April 2018, with descriptive statistics presented in mean and standard deviation, and logistic regression adjusting for confounders.

**Results:** Among the 240 women enrolled in the study, 33% (n= 80) were using supplementary food for their infants at 14 weeks of infants age. Infants who received supplementary feeding at age 14 weeks had nearly 2.5 times higher odds of being wasted (OR: 2.449, *p*-value: 0.002) as compared to exclusively breastfed infants.

**Conclusion:** Infants between 14 to 16 weeks of age who received supplementary feeding were at risk of wasting as compared to exclusively breastfed infants. Despite strong evidence in support of the benefits of exclusive breastfeeding, awareness in urban women in India is low. Increased focus on promoting exclusive breastfeeding is necessary to ensure proper nutritional intake and healthy growth of infants.

## Introduction

The first 6 months of life have higher growth velocity and are also the vulnerable period for nutrition-related health events in infants. Breastfeeding is essential for maintaining the optimal health status of the infants, which includes: providing nutrients, immunity, and improved developmental outcomes (1). It reduces the risk of developing asthma, diabetes mellitus, and obesity (1).

There is strong evidence that breastfed infants are at a reduced risk of being overweight or obese during childhood (2, 3). Evidence from a meta-analysis (4) suggests the risk of overweight was reduced by 4% for every month of breastfeeding -up to 9 months of age. Moreover, infants < 6 months of age who were fed supplementary food were also susceptible to diarrhea, thus leading to weight loss (5). Despite the strong evidence regarding the beneficial effects of breastfeeding, in India, the proportion of exclusive breastfeeding (EBF) rates remain <70% during the first 6 months of age (6, 7) and only 54.2% in the state of Karnataka (urban and rural areas) (8, 9).

Several factors affect initiation, continuation, and cessation of breastfeeding among Indian women. These include physical and psychosocial attributes such as Body Mass Index (BMI), the psychosocial status of the woman during postpartum (up to 6 months after childbirth), age, gestational age, and parity (10–15). Additionally, socio-demographic factors such as education level, socioeconomic status, and sex of the infant (16–18) can affect breastfeeding practices as well. Available evidence on the effect of maternal factors influencing the nutritional status of the infants is limited to infants beyond 6 months. However, most of the evidence has been from cross-sectional studies and thus suffers from limited causal inference. Several other sources of systematic error affect these estimates. As seen in the study by Kerac et al. (19), most studies exclude infants aged < 6 months of age from nutrition surveys resulting in a lack of information on their dietary intake. Applying the growth standards during the early months of life is essential for early detection, preventing poor health and nourishment outcomes as this can affect cognition and physical growth during adulthood (20). Thus, there is a pressing need to prospectively assess the nutritional status of the infants as a result of the feeding practices and other antecedent maternal factors. Thus, we aimed to explore how breastfeeding practices affect the anthropometric status of infants in an ongoing cohort study while assessing the determinants of breastfeeding practices among women in the cohort.

## Methods

### Study Setting and Participants

Maternal antecedents of adiposity and studying the transgenerational role of hyperglycemia and insulin (MAASTHI) is an ongoing 5-year birth cohort study undertaken to prospectively assess the effects of several factors in pregnancy on the risk of adverse maternal and infant outcomes at tertiary care public health facilities in urban Bengaluru, South India. The detailed protocol of MAASTHI has been published earlier (21).

Study participants suitable for the inclusion criteria were approached to participate in the study. The inclusion criteria required that the infants must be between 14 and 16 weeks of age at the time of data collection, born to women who are participants in the MAASTHI cohort study and residing in the study area. The research team did the recruitment after obtaining written informed consent from the participant. The exclusion criteria for the cohort included: the death of the infant, women's history of diabetes, Hepatitis B infection, and HIV seropositivity.

### Sample Size

Since the prevalence of wasted infants is more than overweight infants at 14 weeks of age in India, the prevalence of wasting was used to calculate the sample size. According to the National Family Health Survey round 4 (NFHS-4), the prevalence of wasting in Karnataka is 26% (8). We arrived at a sample size of 200 to detect a difference at 95% confidence interval, with 10% precision and for 80% power (22). We used the final sample size as 240 after accounting for a non-response rate of 20%.

### Data Collection

For this study, data was collected from the eligible participants from April 2016 to April 2018 at the public health facilities and through home visits for those participants who could not come for their follow up visits. Data were collected at three different time points:

1. During the gestational phase - socio-demographic details, psychosocial status, obstetric details and anthropometry of women;
2. Follow up at delivery of women and infants - women's health and psychosocial status, infant health, feeding practices, and anthropometry; and
3. Follow up at 14 weeks post-delivery - women's health and psychosocial status, infant's health, morbidity if any (such as diarrhea), feeding practices, as well as women and infant anthropometry.

### Measurements

The Edinburgh Postnatal Depression Scale (EPDS) is a validated tool to assess psychosocial status, which has been validated in Karnataka as well (23), and social support was measured using the 12- item validated questionnaire (24). For anthropometry of the women, weight was measured using a Tanita weighing scale and height using the SECA 213 Portable Stadiometer, Hamburg. The anthropometry of the infants was measured at birth and 14 weeks; weight using a SECA 354 Weighing Scale, Hamburg; and length using SECA 417 Infantometer, Hamburg.

### Quality Control

All data collection was conducted by experienced and qualified research staff who were trained and certified in anthropometric measurements and nutritional assessments. Stringent quality measures for data collection were maintained throughout by supervision of inter- and intra-observer reliability of measurements assessed by senior team members. Additionally, refresher training and certification was conducted annually. The monthly calibration of all the equipment was done using prescribed guidelines, and a calibration log was maintained that was supervised by the principal investigator (GRB) of the study.

### Data Analysis and Operational Definitions

During data collection, data entry was done on android tablets. Senior team members regularly checked the data entry for errors and missing information. The data collected was exported through MS Excel 2010, and cleaned before analysis.

For this paper, we analyzed the following details: socio-demographic details, women's health, psychosocial status, obstetric details, anthropometry, breastfeeding practices as well as infant anthropometry of the eligible participants from April 2016 to April 2018.

Infants were classified as exclusively breastfed if they were being fed **only breast milk** along with medicines prescribed by the doctor if any. Infants who were fed **breast milk along with formula food/ cow milk/ biscuits/ date syrup** and others were treated as supplementary food fed infants.

Infant feeding practices was the exposure factor, and infant anthropometric parameters (Weight for Length, Weight for Age, and Length for Age) were the outcome factors. **Weight for gestational age percentiles** was calculated based on the recommendations by Kumar et al. (25) for infants of South India. **Weight for Length (WFL), Weight for Age (WFA), and Length for Age (LFA) percentiles** were calculated based on the WHO recommendations for child growth standards (26–28) using percentile charts. Each measurement was categorized as: < 10th percentile – low, from 10th to 90th percentiles - normal and above/more than 90th percentile as high WFL/WFA/LFA. For easier reading, Low WFL is referred to as “**Wasting**,” whereas high WFL can be read as “**Overweight for Length**,” respectively. Similarly, Low WFA and high WFA can be read as synonymous terms as “**Underweight**” and “**Overweight**,” respectively. Low LFA and high LFA can be read as “**Stunting**” and “**Tall for Age**,” respectively.

**Socioeconomic status** was categorized using the Kuppuswamy Socioeconomic Scale 2017 (29). **BMI of the women** during 14 weeks postpartum was classified as per the South Asian criteria (30). A BMI of < 18.5 was classified as underweight, 18.5–22.9 as normal, 23–24.9 as overweight, 25–29.9 as pre-obese, 30–40 as obese type 1, and 40.1–50 as obese type 2. Cut off of **EPDS scores for defining depressive symptoms were** taken as 11 based on our significant findings for the MAASTHI cohort (31).

Chi-Square tests were performed to examine the effect of any potential determinants of the breastfeeding practices of the women in our sample. Based on prior literature, the potential determinants analyzed were BMI of the women (10), EPDS score at 14 weeks (12), age of the women (15), parity (15), gestational age at delivery (15), the educational status of the women and men (16), socioeconomic status (17), sex of infant (18), husbands' smoking status (32), weight for gestational age of infants (33) and social support score at 14 weeks (34). Also, we included confounders based on a review of literature or if the *p* < 0.2 on univariable analysis against the outcome variables (35). Breastfeeding practices were analyzed against the sex of the infant (categorical) and all the confounders (categorical) using binomial regression on Stata.

Descriptive statistics and logistic regression were done using Stata, College Station [Stata/IC 14.2 for Mac (64-bit Intel) Revision 19 Dec 2017, (1985-2015) © StataCorp LLC, StataCorp, 4905Lakeway Dr, College Station, TX] and the chi-square analysis of data was done using the statistical software, R Commander [R x64 3.4.1, R for Windows GUI front-end. R Core Team (36). R a language and environment for statistical computing, R Foundation for Statistical Computing, Vienna, Austria]. We used univariate and multivariate logistic regression to examine the association of the anthropometric parameters of infants (WFL, WFA, and LFA as the outcome variables) against breastfeeding practices (categorical) as an independent variable. **Model 1** consisted of < 10th percentile as the index category and higher than the 10th percentile as the reference category. **Model 2** consisted of higher than 90th percentile as the index and lesser than 90th percentile as the reference category.

### Ethics Consideration

The study was reviewed and approved by the ethics committees at the Indian Institute of Public Health- Gandhinagar (TRC/2017 18/13/12) and Indian Institute of Public Health -Hyderabad (Bangalore Campus) (IIPHHB/TRCIEC/142/2018). The Institutional Ethical Committee (IEC) Board approved the MAASTHI cohort at the Indian Institute of Public Health- Hyderabad (Bangalore Campus). (IIPHHB/TRCIEC/091/2015). The Directorate of Health and Family Welfare, Government of Karnataka has provided approval for conducting the MAASTHI cohort study as well. The study was performed following the relevant guidelines and regulations for public health research. Only women who voluntarily participated and provided written informed consent were enrolled in the MAASTHI cohort study. Participation included data collection during different time points and permission for publication of anonymous data in any report/ journal etc.

## Results

Among the 240 women in the sample population, the mean age of the women was 23.95 ± 3.88 years. The majority of the women (70%; *n* = 169) were in the age category of 18–25 years. About half were Hindus (49%), followed by Muslims (47%). The majority of the women (65.7%) and their husbands (71.1%) had studied until high school. Most of the women were homemakers (95%; *n* = 229), while nearly half of their husbands were unskilled workers (47.9%; *n* = 119). Two- thirds of the women (65.8%; *n* = 158) belonged to Upper-Lower Socioeconomic Class as per the Kuppuswamy classification. None of the women admitted tobacco consumption, but 35.8% (*n* = 86) husbands were smoking tobacco. More than half of the women were primiparous (51.5%; *n* = 123) and 6.6% had preterm deliveries (<37 weeks). (Tables 1, 2).

**Table 1:** Socio-demographic and clinical characteristics of women and infants.

**Characteristic**	**Categories**	**Respondents %**	
		***n* = 240**	
Age of women (years)	18–25	169	70.42
	26–35	68	28.33
	36–45	3	1.25
Religion	Hinduism	118	49.16
	Islam	113	47.08
	Christianity	9	3.75
Women's educational status	Illiterate	6	2.5
	High school	157	65.7
	PUC/diploma	55	23.0
	Graduation/post graduation	21	8.8
Husband's educational status	Illiterate	18	7.5
	High school	170	71.1
	PUC/Diploma	38	15.9
	Graduation/post graduation	13	5.4
Socioeconomic status according to modified Kuppuswamy scale	Upper lower	158	66.1
	Lower middle	67	28.0
	Upper middle	14	5.9
Gestational age at delivery	Pre term	16	6.66
	Term	224	93.33
Parity	Prima	123	51.5
	Multi	116	48.5
BMI of Women at 14 weeks	Underweight	26	10.83
	Normal	85	35.41
	Overweight	39	16.25
	Pre obese	72	30
	Obese class 1	15	6.25
	Obese class 2	3	1.25
HUSBAND'S SMOKING STATUS	Yes	86	35.83
	No	154	64.16
Breastfeeding practice	Exclusive Breastfeeding (EBF)	160	66.66
	Supplementary feeding	80	33.33
EPDS at 14 weeks	Below 11	224	93.33
	11 and above	16	6.66
Social support at 14 weeks	Below 24	46	19.16
	24 and above	194	80.83
Sex of Infant	Male	117	48.75
	Female	123	51.25
Weight for length	<10th (wasting)	91	37.91
	10–90th (normal)	135	56.25
	> 90th (overweight for length)	14	5.83
Weight for age	< 10th (underweight)	82	34.16
	10-90th (normal)	156	65
	> 90th (overweight)	2	0.83
Length for age	< 10th (stunting)	48	20
	10–90th (normal)	175	72.91
	> 90th (tall for age)	17	7.08
Weight for gestational age	Small for Gestational Age (SGA)	30	13.0
	Appropriate for age (AGA)	188	81.7
	Large for gestational age (LGA)	12	5.2
Diarrhea episode	Yes	45	18.8
	No	195	81.3

**Table 2:** Anthropometric measurements of women and infants.

**Characteristic**	**Mean**	**SD**
Age of women (years)	23.954	± 3.882
Weight of women (kg)	56.554	± 11.278
BMI of Women (kg/m^2^)	24.63	± 13.79
Infant weight at birth (kg)	2.851	± 0.512
Gestational age at delivery (weeks)	38.916	± 1.362
Weight of infant at 14 weeks (kg)	5.679	± 0.798
Length of infant at 14 weeks (cm)	60.661	± 3.052
Weight for height	30.94	± 30.849
Weight for age	24.703	± 23.013
Length for age	37.597	± 29.463

At the time of 14 weeks follow up, 66.6% (*n* = 160) were exclusively breastfeeding their infants, while 33.3% (*n* = 80) were feeding their infants supplementary food. The mean BMI of the women at the time of follow up was 24.63 ± 13.79. When assessed for psychosocial status, 6.6% had ≥ 11 EPDS score, indicating depressive symptoms, and 19% (*n* = 46) had poor social support score that was below 24.

For infants born, the sex distribution was almost equal with 48.8% male and 51.2% female infants. About 13% were small for gestational age (12.5%; *n* = 30) and 5% were large for gestational age. At 14 weeks follow up, the mean weight of infants was 5.679 ± 0.798 kg, while the mean length was 60.66 ± 3.05 cm. At least a single episode of diarrhea in infants (very runny, watery stools with mucus and, are at an increased frequency or volume than normal) was reported for almost one-fifth of the infants since birth (19%; *n* = 45) (Tables 1, 2).

Further, to understand the determinants of breastfeeding, we noted that the sex of infants affected the prevalence of supplementary feeding. This was significantly higher among male infants (58.8% in males vs. 41.3% in females) (*p* ≤ 0.05), while the remaining potential determinants showed no effect on breastfeeding (Table 3).

**Table 3:** Determinants of breastfeeding practices.

	**Breastfeeding practices n (%)**		***P*-Value**	***X* squared**	**df**
	**Exclusive breastfeeding**	**Breastfeeding + supplementary feeding**			
**EPDS at 14 weeks**	***N* = 160**	***N* = 80**	0.714	0.134	1
Below 11	150 (93.8)	74 (92.5)			
11 and Above	10 (6.3)	6 (7.5)			
**BMI of women at 14 weeks**	***N* = 160**	***N* = 80**	0.411	5.04	5
Underweight	16 (10.0)	10 (12.5)			
Normal	63 (39.4)	22 (27.5)			
Overweight	27 (16.9)	11 (13.8)			
Pre obese	43 (26.9)	30 (37.5)			
Obese type 1	9 (5.6)	6 (7.5)			
Obese type 2	2 (1.3)	1 (1.3)			
**Sex of infant**	***N* = 160**	***N* = 80**	**0.028**	**4.803**	**1**
Male	70 (43.8)	47 (58.8)			
Female	90 (56.3)	33 (41.3)			
**Age of women (years)**	***N* = 160**	***N* = 80**	0.364	2.021	2
18–25	108 (67.5)	61 (76.3)			
26–35	50 (31.3)	18 (22.5)			
36–45	2 (1.3)	1 (1.3)			
**Women's education status**	***N* = 159**	***N* = 80**	0.791	1.041	3
Illiterate	4 (2.5)	2 (2.5)			
High school	101 (63.5)	56 (70.0)			
PUC/Diploma	39 (24.5)	16 (20.0)			
Graduation	15 (9.4)	6 (7.5)			
**Men's educational status**	***N* = 159**	***N* = 80**	0.721	1.333	3
Illiterate	11 (6.9)	7 (8.8)			
High School	115 (72.3)	55 (68.8)			
PUC/Diploma	26 (16.4)	12 (15.0)			
Graduation	7 (4.4)	6 (7.5)			
**Socioeconomic status**	***N* = 159**	***N* = 80**	0.978	0.044	2
Lower middle class	105 (66.0)	53 (66.3)			
Upper lower class	45 (28.3)	22 (27.5)			
Upper middle class	9 (5.7)	5 (6.3)			
**Social support**	***N* = 160**	***N* = 80**	0.908	0.013	1
Below 24	31 (19.4)	15 (18.8)			
24 and above	129 (80.6)	65 (81.3)			
**Parity**	***N* = 159**	***N* = 80**	0.11	2.555	1
Prima	76 (47.8)	47 (58.8)			
Multi	83 (52.2)	33 (41.3)			
**Men's smoking status**	***N* = 160**	***N* = 80**	0.128	2.32	1
Yes	52 (32.5)	34 (42.5)			
No	108 (67.5)	46 (57.5)			
**Weight for gestational age**	***N* = 153**	***N* = 77**	0.41	1.785	2
Appropriate for gestational age	127 (83.0)	61 (79.2)			
Small for gestational age	17 (11.1)	13 (16.9)			
Large for gestational age	9 (5.9)	3 (3.9)			
**Gestational age at delivery**	***N* = 160**	***N* = 80**	0.36	0.837	1
pre term	9 (5.6)	7 (8.8)			
Term	151 (94.4)	73 (91.3)			

*The bold indicates statistically significant values*.

Logistic regression showed that feeding practice had an effect on weight for length percentiles (WFL) (for measuring wasting) and weight for age percentiles (for measuring underweight) of infants. Infants that were fed breast milk along with supplementary food had nearly 2.5 times higher odds of wasting (OR: 2.449, *p*-value: 0.002, 95% CI: 1.393–4.304) and 1.8 times higher odds of underweight for age (OR: 1.803, *p*-value: 0.039, 95% CI: 1.029–3.159) as compared to exclusively breastfed infants.

After adjusting for potential confounders, similar results were noted for WFL percentiles, with the odds increasing by a mere 0.06 times; however, odds for the weight for age percentiles remained the same (Table 4).

**Table 4:** Association between infant feeding practices and anthropometry of infants using logistic regression.

**Status of nutrition**	**Outcome**		**Odds Ratio**	**95% C.I**.		***p*-value**
				**LL**	**UL**	
Undernutrition^*^	Weight for length (wasting)	Crude	**2.449**	**1.393**	**4.304**	**0.002**
		Adjusted	**2.561**	**1.379**	**4.753**	**0.003**
	Weight for age (underweight)	Crude	**1.803**	**1.029**	**3.159**	**0.039**
		Adjusted	1.817	0.992	3.326	0.053
	Length for age (stunting)	Crude	1.135	0.581	2.220	0.711
		Adjusted	0.894	0.438	1.827	0.759
Overweight- obesity^**^	Overweight for length	Crude	0.779	0.205	2.956	0.714
		Adjusted	0.607	0.143	2.576	0.499
	Overweight	Crude	73,430	0		0.996
		Adjusted	2.292E	0		0.994
	Tall for Age	Crude	1.129	0.398	3.207	0.820
		Adjusted	0.922	0.294	2.890	0.889

* *Models assessing undernutrition: < 10th percentile- Index category, > 10th percentile is the reference category*.

** *Models assessing over nutrition: > 90th percentile- Index category, < 90th percentile is the reference category*.

*All the regression models have been adjusted for potential confounders like age, educational status of women, socioeconomic status, BMI of women, husband's smoking status, parity, gestational age at delivery, sex, EPDS score, and social support score at 14 weeks, and weight for gestational age*.

*The bold indicates statistically significant values*.

We also found that male infants had higher odds of being fed supplementary food in unadjusted analysis. They had nearly 1.8 times higher odds of being fed supplementary food (OR: 1.831, *p*-value: 0.029, 95% CI: 1.063–3.154) when compared to female infants. However, the role of sex on breastfeeding practices disappeared after adjusting the confounders (Appendix 1).

## Discussion

India is currently facing a double burden of undernutrition and obesity (37). Proper nutritional intake is thus essential during the early months of life to tackle this double burden (38–42). Breastfeeding deemed as the ‘gold standard’ as early as 1981 by Kramer et al. (43), showed that optimal nutrition during the first hour of birth and subsequent 6 months plays a vital role in child survival, health, and development (44). Further, it also aids in improving the emotional and mental health of the mother, along with the added economic benefits (45, 46). However, in low-middle-income countries like India, supplementary feeding of infants is initiated early (6, 7, 47), which may contribute to poor feeding practices that in turn is attributed to under-5 morbidity and long term health implications (2, 3, 5, 20). According to Barker et al. undernutrition starting right from the *in-utero* stage can put the individual at risk of being overweight and having non-communicable diseases (NCDs) (48). There is also evidence to show that children with lower BMI have early adiposity rebound (49, 50). Similarly, other epidemiological studies have found that smaller size at birth, and infancy is associated with increased rates of cardiovascular diseases, Type 2 diabetes mellitus (T2DM), adiposity, and metabolic issues (51–54). Recent research has also shown that the gut microbiota can be modified by early nutrition, formula feeding can lead to early life dysbiosis and development of NCDs (55).

Despite massive awareness regarding the benefits of breastfeeding, according to the recent *Poshan Abhiyan* report that mentions that less than two-thirds of infants are breastfed across the country and Karnataka state (56). The numbers are further reduced during the following months, as many women are likely to discontinue breastfeeding and initiate supplementary feeding after 3 to 4 months due to numerous reasons (57). In our study, some women initiated supplementary feeding as early as the 1st month itself, while the majority initiated after 3 months of age. However, according to a recent study in rural Karnataka, 61% of infants were exclusively breastfed even at 4 months age (58) which is similar to our study findings as assessed during follow-up at 14 weeks of infant age.

We also found a significantly higher proportion of supplementary feeding in urban Indian male infants. This could perhaps be attributed to the rampant advertising of supplementary milk/feed in the markets. Mothers or family members might perceive that supplementary food might be superior or might help compared to breastfeeding. They are more likely to be fed formula food and weaned earlier (18) with a perception that it would make them healthy and active. This reflects in our study as well; the male infants had a higher prevalence of formula feeding when compared to female infants. Although we did not find statistically significant results for the association of sex with breastfeeding practices upon adjusting for potential confounders, the higher proportion of supplementary feeding in male infants is a concerning practice. In a study conducted by Angadi et al. (59) in Karnataka, no significant difference in breastfeeding practices was found among the sexes.

Additionally, though some studies showed an association, regarding education levels and social class (16, 17), our findings showed no association concerning breastfeeding and awareness especially among lesser educated and lower socioeconomic class parents. We also noted that wasting was reported in almost 38% of infants (using the WHO standards of < 10th WFL percentile), which was relatively higher than the state estimates according to NFHS-4 (60). This contradictory finding perhaps could be due to different standards for measuring wasting in infants and methodological variations.

Another important finding from our study revealed that infants who were both breast- and fed with supplementary foods at 14 weeks of age had a significant association of wasting with nearly 2.5 times higher odds as compared to EBF infants, after adjusting for confounders. The use of supplementary food before one year perhaps could attribute to wasting among other factors and accelerate wasting in infants. Thus, bearing in mind the multifactorial causes of malnutrition and subsequent obesity in childhood, cessation of breast milk and sole use of supplementary feed must be avoided. Moreover, as recommended by the WHO, the practice of exclusive breastfeeding for the first 6 months, followed by a timely introduction of complementary food along with breast milk, must be practiced (61).

Further, we presented evidence for EBF and the need for starting the supplementary feeding as late as possible. Mostly this might be due to reported episodes of diarrhea among infants that were fed with supplementary foods. Corroboration for the same has been supported by earlier studies (5, 6) that suggest the use of unsterilized water or utensils to prepare the feed may cause contamination, thus resulting in diarrhea due to the infant's underdeveloped immune system. This, in turn, may lead to low weight-for-length percentiles among fed with supplementary foods infants, as seen in our study as compared to EBF infants. Cow milk, formula food mixed with water and, biscuits dipped in water were the most common types of supplementary food fed to the infants. Cow milk has relatively higher levels of protein and casein compared to human milk, which can be difficult for an infant to digest (62). Also, it is difficult to ascertain if the water used for infant feeding was sterile since most of the participants were from the lower socioeconomic class.

Despite substantial evidence (12–14, 23) on psychosocial status affects breastfeeding, no significant association between EPDS scores and breastfeeding practices were noted in our study. Similarly, although studies suggesting that women with obesity had difficulty in breastfeeding and ceased breastfeeding earlier (10, 11, 63), BMI of the women in our study and breastfeeding practices were not affected. Again this could be attributed to the South Asian criteria to classify the BMI used in our study. Further studies also suggest that preconception BMI may affect infant weight gain (10, 64). However, our cohort was limited to the estimation of BMI after conception/during gestation only. Despite the limitation, dissemination of the results of this study may further validate the importance of breastfeeding and lead to increased emphasis on tackling childhood nutritional problems.

Early childhood feeding practices widely influence child growth and survival. Undernutrition has been strongly related to disability and infant mortality, while the growing burden of obesity poses significant risks during adulthood. Hence, applying growth standards for detecting poor growth during the early months of life is essential for preventing poor health and nourishment outcomes in the infant's first year. Therefore, findings from our study present immense public health implications and can be used for further policy action and change. The importance of breastfeeding and the ill-effects of early cessation, coupled with complementary feeding, are lessons learned that should be applied to aid in tackling childhood nutritional problems. Policy at the state and national level must address the need for multi-sectoral approaches. These should focus on overcoming barriers associated with breastfeeding during the first year of the infant's life by lactation consultants, doctors, health workers; and promote conducive environments by spouses, family members, and social support.

In summary, our findings indicate that infants between 14 and 16 weeks of age who received supplementary feeding were at risk of wasting as compared to exclusively breastfed infants. Despite strong evidence in support of the benefits of exclusive breastfeeding, the awareness levels regarding this are not very high in the cohort. However, we could not find any conclusive evidence that any of the potential determinants had an effect on breastfeeding practices. Early childhood feeding practices widely influence child growth and survival. Increased focus on promoting exclusive breastfeeding during the first 6 months of life is necessary to ensure proper nutritional intake and healthy growth of infants. We must reflect upon the socio- cultural and systemic barriers and, the community's perspectives regarding breastfeeding to bring about effective awareness in the society.

## Data Availability Statement

The datasets generated for this study are available on request to the corresponding author.

## Author Contributions

SKo conceptualized the study, collected certain sections of the data, analyzed the results, and wrote the manuscript. GB provided conceptual guidance, analyzed the data, and wrote certain sections of the manuscript. DR was involved in the conceptualization of the study, collected certain sections of the data, and edited the manuscript. VI provided conceptual guidance and edited the manuscript. AY and PS collected individual sections of the data and edited the manuscript. EL edited and wrote individual sections of the manuscript. SKi and GM provided guidance and edited the manuscript. All the authors have reviewed and approved the final version of the manuscript.

## Conflict of Interest

The authors declare that the research was conducted in the absence of any commercial or financial relationships that could be construed as a potential conflict of interest.

## Abbreviations

AGA: Appropriate for Gestational Age
BMI: Body Mass Index
EBF: Exclusive Breastfeeding
EPDS: Edinburgh Postnatal Depression Scale
LGA: Large for Gestational Age
LFA: Length for Age
MAASTHI: Maternal antecedents of adiposity and studying the transgenerational role of hyperglycemia and insulin
NCDs: Non Communicable Diseases
NFHS: National Family Health Survey
ROC: Receiver Operating Characteristic
SGA: Small for Gestational Age
T2DM: Type 2 Diabetes Mellitus
TRC, IEC: Technical Review Committee-Insti Organization.
