# Supplementary material for: Determinants of Breastfeeding Practices and Its Association With Infant Anthropometry: Results From a Prospective Cohort Study in South India
Source: Front Public Health. 2020 Oct 14;8:492596. doi: 10.3389/fpubh.2020.492596 (PMC7116249; doi:10.3389/fpubh.2020.492596)
Supplement: Supplementary file 1 [file Table_1.DOCX]

**Appendix 1**

Association between sex of infants and breastfeeding practices using logistic regression

| **Outcome of Interest** |  | **Odds Ratio** | **95% C.I.**  **LL UL** | **p- Value** |
| --- | --- | --- | --- | --- |
| **Breastfeeding Practices** | Crude | **1.831** | **1.063 3.154** | **0.029** |
|  | Adjusted | 1.793 | 0.982 3.272 | 0.057 |

Model adjusted for: age, educational status of women and men, socioeconomic status, BMI of women, husband’s smoking status, parity, gestational age at delivery, EPDS score at 14 weeks, social support score at 14 weeks and religion.

Association between sex of infants and anthropometry of infants using logistic regression

| **Outcome** |  | **Odds Ratio** | **95% C.I.**  **LL UL** | **p-value** |
| --- | --- | --- | --- | --- |
| **Weight for Length (Wasting*)** | Crude | **2.594** | **1.500 4.484** | **0.001** |
|  | Adjusted | **2.487** | **1.387 4.457** | **0.002** |
| **Weight for Age (Underweight*)** | Crude | **1.827** | **1.063 3.140** | **0.029** |
|  | Adjusted | **1.936** | **1.086 3.450** | **0.025** |
| **Length for Age (Stunting*)** | Crude | 1.291 | 0.681 2.447 | 0.434 |
|  | Adjusted | 1.307 | 0.668 2.556 | 0.434 |

Model adjusted for: age, educational status of women, socioeconomic status, EPDS score at 14 weeks, social support score at 14 weeks, weight for gestational age and breastfeeding practices.
